# Crocetin Isolated from the Natural Food Colorant Saffron Reduces Intracellular Fat in 3T3-L1 Adipocytes

**DOI:** 10.3390/foods9111648

**Published:** 2020-11-12

**Authors:** Elena Jiménez-Ortega, Aitana Braza-Boïls, Miguel Burgos, Natalia Moratalla-López, Manuel Vicente, Gonzalo L. Alonso, Eduardo Nava, Sílvia Llorens

**Affiliations:** 1 Department of Crystallography and Structural Biology, Institute of Physical-Chemistry Rocasolano, CSIC, 28006 Madrid, Spain; xelena@iqfr.csic.es; 2 Unidad de Cardiopatías Familiares, Muerte Súbita y Mecanismos de Enfermedad (CaFaMuSMe), Instituto de Investigación Sanitaria La Fe, 46026 Valencia, Spain; aitana_braza@iislafe.es; 3 Translational Research Unit, Albacete University Hospital, 02008 Albacete, Spain; miguel.burgos@uclm.es; 4 Cátedra de Química Agrícola, ETSI Agrónomos y de Montes, Universidad de Castilla-La Mancha, Campus Universitario, 02071 Albacete, Spain; Natalia.Moratalla@uclm.es (N.M.-L.); Gonzalo.Alonso@uclm.es (G.L.A.); 5 Department of Medical Sciences, Faculty of Medicine of Albacete, Centro Regional de Investigaciones Biomédicas (CRIB), University of Castilla-La Mancha, 02008 Albacete, Spain; Manuel.Vicente@alu.uclm.es (M.V.); eduardo.nava@uclm.es (E.N.)

**Keywords:** saffron, crocetin, obesity

## Abstract

Saffron, as a food colorant, has been displaced by low-cost synthetic dyes. These have unhealthy properties; thus, their replacement with natural food colorants is an emerging trend. Obesity is a worldwide health problem due to its associated comorbidities. Crocetin esters (crocins) are responsible for the red saffron color. Crocetin (CCT) exhibits healthful properties. We aimed to broaden the existing knowledge on the health properties of CCT isolated from saffron, to facilitate its consideration as a healthy natural food colorant in the future. We evaluated the ability of CCT (1 and 5 μM) to reduce lipid accumulation during the differentiation of 3T3-L1 preadipocytes. Intracellular fat was quantified by Oil Red O staining. CTT cytotoxicity was measured using the 3-(4,5-dimethylthiazol-2-yl)-2,5-diphenyltetrazolium bromide (MTT) assay. The number and size of lipid droplets were analyzed using WimLipid software. The expression of adipogenic genes (CCAAT/enhancer-binding protein (C/EBPβ, C/EBPδ, C/EBPα), and peroxisome proliferator-activated receptor γ (PPARγ)) was analyzed using quantitative real-time PCR (qRT-PCR). CCT 5 μM decreased intracellular fat by 22.6%, without affecting viability or lipid droplet generation, via a decrease in C/EBPα expression, implicated in lipid accumulation. Thus, CCT is a potential candidate to be included in dietary therapies aimed at reversing adipose tissue accumulation in obesity.

## 1. Introduction

Additive colorants are found in large quantities in food because consumers often associate them with the flavor, safety, and nutritional value of the foodstuff, thus making it more attractive [1]. The food industry uses synthetic food colorants to a large extent, mainly due to their low cost and high stability [2]; however, most of them contain azo functional groups and aromatic ring structures, which can be harmful to human health [3], with additional environmentally harmful effects during food processing [4]. Thus, an acceptable daily intake (ADI) of authorized food additives is continuously evaluated by regulatory bodies, such as the European Food Safety Authority (EFSA) in Europe and the Food and Drug Administration (FDA) in the United States (US), to adequately protect consumers [5]. Even so, consumers remain cautious with regard to the safety of synthetic dyes, whereas they are also aware that many natural colorants provide health benefits; thus, the replacement of artificial food colorants with natural ones is a current market demand [2].

Naturally occurring color additives from vegetable and mineral sources were used to color foods, drugs, and cosmetics in ancient times. Paprika, turmeric, and saffron are some examples obtained from vegetables. From the second half of the 19th century onward, artificial colors rapidly replaced natural colorants. Artificial colorants are used in a wide variety of foods, mainly to make them more attractive to consumers; in fact, their intake by consumers has increased since 1950 [2]. Currently, consumers prefer natural colorants in foodstuff or for cookery since almost all of them are hypoallergenic and nontoxic, while displaying salutary properties in humans, which is a significant advantage over many artificial dyes. In addition, their production, use, and elimination is environmentally friendly and can contribute to sustainable development [6]. The current market trend involves the replacement of artificial dyes with natural ones [2].

The most important use of saffron (*Crocus sativus* L.) is in cookery; its dried stigmas constitute the saffron spice. This spice is greatly valued for its coloring, flavoring, and aromatizing properties in some traditional dishes, as well as in modern cuisine. In addition to its use as a spice, saffron has long been considered a medicinal plant for its therapeutic properties [7]. However, for years, the use of this spice as a food colorant, due to its high price, has been replaced by low-cost synthetic dyes (e.g., tartrazine (TTZ)) [8]. The consumption of TTZ can produce adverse metabolic effects [3,9,10,11]. Indeed, TTZ has been banned in some countries including Norway and Austria [12]. Turmeric (whose main coloring component is curcumin) has excellent heat stability, and it is often used as a replacement for TTZ; however, this pigment is unstable when exposed to light and it is susceptible to oxidation [2]. In contrast, saffron pigments are quite light- and heat-resistant [13].

Saffron contains several bioactive compounds, of which crocins (crocetin esters), a group of water-soluble carotenoids derived from crocetin (CCT, the aglycon of crocin), are responsible for the intense color that saffron provides to aqueous solutions [14]. Saffron displays numerous functional and bioactive properties. Therefore, research into the effects of saffron and its components is necessary to achieve a more widespread use of the spice. Today, along with the current trend of using natural colorants, there is a growing interest in therapeutic diets, which include culinary herbs or spices to support therapies for chronic diseases, including obesity [14]. Obesity is a global health problem that is acquiring an enormous epidemiological relevance due to its increasing prevalence rate [15]. The World Health Organization defines obesity as an abnormal or excessive accumulation of fat (adiposity) that can be harmful to health, and it is a risk factor for diabetes, cardiovascular disease, and cancer [16].

Recently, the most important therapeutic effects of saffron were attributed to CCT in its free-acid form [17]. Typical carotenoids contain 40 carbon atoms (C40); however, CCT is a C20 apocarotenoid (C_20_H_24_O_4_; molecular weight 328.4 g/mol), and it is generated via the hydrolysis of crocin glycosides. Crocin is crocetin digentiobiase ester, whereas CCT is 8,8′-diapo-ψ,ψ′-carotenoic acid. CCT contains a carboxyl group at each end of the polyene chain; when ionized, it can function as an acid (anionic) dye for biological staining [18]. On the other hand, CCT has high antioxidant power and possesses a wide range of beneficial properties for humans including anti-inflammatory, antiatherosclerotic, antihypertensive, and anticancer activities [19,20,21,22].

Adipose tissue function is essential for health; it is pivotal in the synthesis and storage of triacylglycerol in lipid droplets (lipogenesis) and the release of fatty acids into systemic circulation during periods of scarcity. In addition, adipocytes are a source of numerous proteins and hormones with actions relevant in practically every aspect of human physiology, including cardiovascular physiology. A crucial process for the homeostatic maintenance of lipid metabolism is the generation of new adipocytes from preadipocytes, a process known as adipogenesis. The process of adipogenesis involves growth arrest, mitotic clonal expansion, early differentiation, and terminal differentiation [23]. In vitro, adipogenesis takes place in two sequential stages: (1) the early stage, dependent on the activation of early transcription factors: CCAAT/enhancer-binding protein (*C/EBP*)*β* and *C/EBPδ*, which in turn activate the transcription factors of the (2) late stage, dependent on the activation of late genes: *C/EBPα* and peroxisome proliferator-activated receptor γ (*PPARγ*) [24,25,26]. In this way, preadipocytes differentiate into an adipocytic phenotype, causing morphological changes in the cell, including lipogenesis [27]. An exquisitely accurate adipogenesis process preserves lipid health [28], with increased lipid accumulation caused by an altered adipogenic process being a key factor in obesity. Thus, intervention in the regulation of adipogenesis, in terms of reducing fat mass, has been proposed as a possible therapy to prevent adipose tissue development and obesity [29]. In this sense, several studies have shown that CCT could play a preventive or even therapeutic role in some aspects related to the comorbidities that accompany obesity. Indeed, CCT has been shown to prevent visceral fat accumulation and insulin resistance induced by a hypercaloric diet in rats [30]. In addition, CCT regulates the expression of adiponectin in the adipose tissue of fructose-fed rats [31].

We observed in previous studies that different components of saffron, such as CCT and crocins, on some occasions, have opposite vasoactive properties [21]. Therefore, during this trend of a change toward healthier and more sustainable natural products, coinciding with the rapid advance of obesity in the world, we aimed to study CCT isolated from saffron (*C. sativus* L.) to broaden the existing knowledge on its beneficial properties and to promote its use as a healthy natural food colorant in the future. Specifically, our aim was to test the ability of CCT to reduce adipocytic lipid accumulation. We examined the ability of the CCT to induce differentiation in cultured murine 3T3-L1 preadipocytes by studying the amount of intracellular fat, the number and size of lipid droplets, and the viability and expression of the main early (*C/EBPβ* and *C/EBPδ*) and late (*C/EBPα* and *PPARγ*) genes involved in differentiation from preadipocytes to adipocytes. CCT decreased intracellular fat in mature adipocytes, showing potential antiadipogenic properties. Additionally, CCT did not affect lipid droplet generation or cellular viability. On the other hand, we report here that CCT diminished the messenger RNA (mRNA) levels of the transcription factor *C/EBPα*, which is implicated in lipid accumulation. Therefore, we propose that CCT reduces intracellular fat by decreasing *C/EBPα* mRNA levels.

## 2. Materials and Methods

### 2.1. Plant Material and Isolation of CCT

Saffron was obtained from the “Agrícola Técnica de Manipulación y Comercialización” company (Minaya, Albacete, Spain) during the 2014–2015 harvest. These dried stigmas belonged to the Protected Designation of Origin (PDO) “Azafrán de La Mancha”, which complies with ISO 3632:2011 (Category I) and guarantees their origin and freedom from fraud. Saffron with a very low moisture level was stored in the dark at 4 °C until further use.

CCT was obtained via the hydrolysis of aqueous solutions of saffron acquired using a protected internal method of the “Verdú Cantó Saffron Spain” company (Novelda, Alicante, Spain) [22]. CCT purity was checked through the reverse-phase (RP)-HPLC–diode array detection (DAD) technique according to [32]. Twenty microliters of aqueous extracts of CCT were filtered through a syringe with a polytetrafluoroethylene (PTFE) filter, 0.45 µm pore size (Millipore, Bedford, MA, USA), and injected into an Agilent 1200 chromatograph (Palo Alto, CA, USA). Chromatographic determination was achieved using a Phenomenex Luna C18 column (150 × 4.6 mm, 5 μm) (Le Pecq CEDEX, France) equilibrated at 30 °C. Acetonitrile (ACN) and Milli Q water (mQW) were used as the mobile phase at a flow rate of 0.8 mL/min. HPLC-grade ACN was obtained from Panreac^®^ (Barcelona, Spain) and ultrahigh-purity water mQW was produced using a Milli-Q system (Millipore, Danvers, MA, USA). The elution gradient was set up for the ACN solvent as follows: 20%, 0–5 min; 20–80%, 5–15 min; 80%, 15–18 min; and 20%, 18–30 min. The DAD detector (Hewlett Packard, Waldbronn, Germany) was set to 440 nm for *cis*/*trans*-CCT detection. The chromatographic purity of *cis*/*trans*-CCT according to HPLC–DAD at 440 nm was 99% (86% *trans*-CCT, retention time: 16.64 min; 13% *cis*-CCT, retention time: 17.86 min). CCT was stored at −20 °C until further use. Before using CCT to carry out the experiments, purity was determined again obtaining the same chromatographic purity before it was employed.

### 2.2. 3T3-L1 Cell Culture and Adipocyte Differentiation

**Figure 1:**
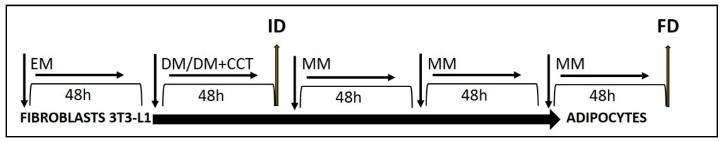
Schematic representation of the differentiation procedure to analyze the effects of crocetin (CCT). Fibroblasts achieved confluence after 48 h of treatment with expansion medium (EM). Then, the differentiation of confluent fibroblasts was induced with differentiation medium (DM; with or without CCT (1 or 5 μM)) for 48 h. Subsequently, preadipocytes were maintained with maintenance medium (MM) for 6 days. ID, initial differentiation; FD, final differentiation.

The cell line of embryonic fibroblasts, 3T3-L1, was acquired in 2016 from the ATCC (American Type Culture Collection, Manassas, VA, USA). 3T3-L1 preadipocytes were cultured, maintained, and differentiated according to the supplier’s instructions. In all experiments, cells were used within the sixth passage. Briefly, 3T3-L1 cells were expanded in a 75 cm^2^ flask at 37 °C under a humidified 5% CO_2_ atmosphere in preadipocyte expansion medium (EM; Dulbecco’s modified Eagle’s medium (DMEM, 90%) supplemented with l-glutamine (1%), penicillin/streptomycin (0.5%), and inactivated bovine calf serum (BCS, 10%). When the cells reached 70–80% confluence, they were seeded on six- or 96-well sterile plates and grown in EM for 48 h or until the culture reached 90% confluence. Then, differentiation of adipocytes was induced in the absence (as a control of differentiation) or presence of CCT (1 or 5 μM). Stimulating and inhibiting controls of differentiation were also established with rosiglitazone (10 μM, an agonist of PPARγ that activates adipogenesis [33,34]) or genistein (12.5 μM, an isoflavone that inhibits adipogenesis [35,36]), respectively. Genistein at this concentration inhibits lipid accumulation while preserving the viability of preadipocytes [36]. For this, post-confluent cells were treated for 48 h with differentiation medium (DM). The DM was prepared using the same components as the EM, instead replacing CBS with inactivated fetal bovine serum (FBS, 10%) and adding an adipogenic cocktail (AC), containing substances to induce differentiation (0.5 mM 3-isobutyl-1-methylxanthine (IBMX), 0.25 μM dexamethasone, and 1 μg/mL insulin (INS)). The DM was subsequently replaced with adipocyte maintenance medium (MM), which was composed of DMEM containing 1 μg/mL INS and 10% FBS. Cells were maintained in MM for 6 days, with the medium replenished every 2 days. At this point, the cells developed large lipid droplets and were considered mature adipocytes (Figure 1).

To check the effect of CCT on the early and late genes of differentiation, cells were collected at two time-points: 48 h after induction (initial differentiation, ID) and 6 days after differentiation (final differentiation, FD).

Two concentrations of CCT (1 and 5 μM) were tested in this work. This choice was based on our experience in previous studies [22] and on the work of Chryssanthi et al. [37]. These were prepared from a stock CCT solution, dissolved in sterile dimethyl sulfoxide (DMSO) and added to DM (the final concentration of DMSO in the culture medium was 0.001% (*v/v*)). Rosiglitazone and genistein stock solutions were also prepared in DMSO and added to the DM to reach a working concentration (the final volume of DMSO in the well was 0.001% (*v/v*)). The differentiation of adipocytes was also carried out in the presence of sterile DMSO (0.001%) as the solvent control. DMSO is usually well tolerated with no observable toxic effects on cells at a 0.1% final concentration. This compound is widely used as a solvent for various pharmacological agents at concentrations of 0.05–1.5% [38].

### 2.3. Quantification of the Intracellular Fat by Oil Red O Staining

Cells differentiated in 96-well sterile plates (cellular density, 4 × 10^3^ cell/well) were stained with Oil Red O (OR) at the FD time-point, according to the method developed by Kraus and colleagues [39] with slight modification. Oil Red O is a dye that strongly stains lipids, specifically triacylglycerol, often used for the quantitative analysis of adipocyte differentiation [39].

The OR stock solution was prepared the day before as follows: 0.2 g OR was dissolved in 100 mL of isopropanol for 24 h at room temperature under agitation. The OR working solution was prepared by mixing six parts of OR stock solution and four parts of double-distilled water (ddH_2_O). The solution was filtered through a two-layer Whatman paper to remove any precipitate.

To stain cells with the OR working solution, they were first washed three times with phosphate-buffered saline (PBS) and fixed in 4% formaldehyde for 1 h at room temperature while avoiding any shaking of the plate. Formaldehyde was removed, and the cells were washed once with cold PBS and air-dried for 10 min. The freshly prepared OR working solution was added to the plates to cover the cell surface. After 10 min, the solution was aspirated, and the cells were washed three times with cold PBS and air-dried for 15 min. OR was eluted with 100% isopropanol for 10 min, and absorbance was measured using a spectrophotometer (ASYS UVM 340, Cambridge, United Kingdom, Microplate Readers) at 450 nm. Data obtained from at least 10 replicates of each condition from three independent experiments were used for analysis. The amount of color produced is directly proportional to the amount of intracellular fat.

Reagents, unless specified otherwise, were acquired from Sigma-Aldrich.

### 2.4. Determination of the Number and Size of Lipid Droplets

The number and size distribution of lipid droplets were evaluated by Wimasis (Edificio Centauro, 14014 Córdoba, Spain) using a WimLipid image analysis software. For this, photomicrographs (20X) of the wells at the FD time-point were taken using a phase-contrast microscope (Olympus 1X51). Parameters such as the circularity, convexity, and elongation were included in the analysis to discriminate drops. The following criteria were used: area ≥ 10 pixels (Px); circularity > elongation; convexity > 0.95. Drops that failed to meet these criteria were removed.

### 2.5. Quantification of Cellular Viability

#### 2.5.1. 3-(4,5-Dimethylthiazol-2-yl)-2,5-diphenyltetrazolium bromide (MTT) Assay

To perform the 3-(4,5-dimethylthiazol-2-yl)-2,5-diphenyltetrazolium bromide (MTT) assay, cells were grown on 96-well sterile plates at a cellular density of 4 × 10^3^ cell/well. The MTT viability assay was carried out as previously described [40] with slight modification. The MTT assay measures mitochondrial activity in metabolizing cells and, therefore, can be used as an approximate measurement of cell viability. The assay relies on the reduction of MTT, a yellow water-soluble tetrazolium dye, primarily by mitochondrial dehydrogenases, to purple-colored formazan crystals. An MTT stock solution in PBS was freshly prepared and assessed in all experimental conditions. At the FD time-point, cells were washed with red phenol-free DMEM without FBS. Then, 100 μL of MTT solution (red phenol-free DMEM with MTT 0.5 μg/μL) was added to each well, mixed gently, and incubated for 45 min at 37 °C. Media were immediately aspirated and discarded; then, in order to solubilize formazan crystals, 100 μL of DMSO was added to each well before gently stirring for 3–5 min. Absorbance was determined spectrophotometrically at 570 nm using a reference wavelength of 630 nm (ASYS UVM 340, Cambridge, United Kingdom, Microplate Readers). The color intensity is directly proportional to the number of viable cells. Data obtained from at least 10 replicates in each experimental condition from three independent experiments were used for analysis. Absorbance was measured for wells containing the control differentiated cells (DM-differentiated cells) and the DM + CCT (1 or 5 μM)-differentiated cells. Furthermore, differentiated cells in the presence of solvent (DM + DMSO (0.001%)) and an activator (DM + rosiglitazone 10 μM) or inhibitor (DM + genistein 12.5 μM) of adipogenesis were also measured.

#### 2.5.2. Trypan Blue Assay

The MTT assay detects viable cells but does not take into consideration cell loss caused by cell death; thus, the percentage of viable cells was additionally determined using a dye exclusion test with trypan blue (TB) dye, which is based on the principle that living cells possess intact cell membranes that exclude TB, whereas dead cells do not. The TB test was performed as previously described [41]. Briefly, an aliquot of cell suspension was centrifuged (950 rpm during 5 min, Eppendorf Centrifuge 5804, Hamburg, Germany), and the pellet was resuspended in PBS or serum-free complete medium. Then, 10 μL of cell suspension corresponding to each experimental condition was mixed with 10 μL of TB (0.4%). After incubating the mixture for 3 min at room temperature, the cells were examined using an automated cell counter (TC10TM, Hercules, CA, USA, BioRad).

### 2.6. Expression of Main Genes Related to Early and Late Differentiation

To analyze the expression of early and late genes, cells were collected at the ID and FD time-points, respectively. Total RNA was extracted from cells differentiated in sterile six-well plates (at a cellular density of 8 × 10^4^ cell/well) using the extraction kit PureLink™ RNA Mini Kit (Waltham, MA USA, ThermoFisher Scientific), according to the manufacturer’s instructions. The extracted RNA was verified and quantified spectrophotometrically using NanoDrop (Thermo Scientific). Complementary DNA (cDNA) was synthesized from 1 μg of RNA using the RevertAid H Minus First-Strand cDNA synthesis kit (Fisher Scientific), according to the manufacturer’s protocol. Gene expression was assessed using quantitative real-time PCR (qRT-PCR) in a LightCycler 480 II thermocycler with Fast Sybr Green Master Mix (Waltham, MA USA, Applied Biosystem). β-Actin was used as an endogenous control. The primer sequences used for amplification are presented in Table A1 (Appendix A).

The reaction mixtures were incubated for an initial denaturation at 95 °C for 10 min, followed by 45 PCR cycles (95 °C for 15 s, 60 °C for 1 min, 95 °C for 15 s, and 60 °C for 1 min). The ΔΔCT method was used to measure relative quantification, and the levels of transcripts were normalized to that of β-actin. The levels of each mRNA were calculated as relative expression to the basal condition (designated as 1). 3T3-L1 cells treated for 48 h with the AC, without CCT, were considered representative of the basal condition. Three independent experiments were performed, each in duplicate.

Calibrated ΔCt values from undifferentiated and control differentiated cells were used to evaluate the expression of genes. Fold changes in gene expression were calculated using the 2^−ΔΔCt^ method [28]. Expression of the *aP2-1* gene was used as a specific adipocyte marker. *aP2* is widely used as a marker of differentiated adipocytes [42].

### 2.7. Data Analysis

All data were presented as the mean ± SD. One-way ANOVA and a post hoc Bonferroni’s multiple-comparison test, using GraphPad Prism version 5.0 software, were used to identify differences between groups. The results were considered to be significant at a *p*-value < 0.05.

## 3. Results

### 3.1. Crocetin Reduced Intracellular Fat in 3T3-L1 Adipocytes

**Figure 2:**
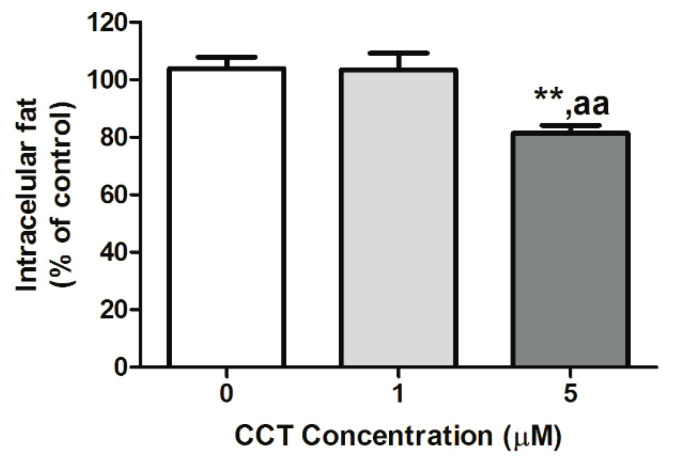
Effect of CCT on lipid accumulation. The maximum intracellular fat detected in control differentiated cells (CCT 0 μM) was set to 100%; the relative intracellular fat levels of CCT-differentiated cells are shown. Values were obtained at the FD time-point. Data from at least 10 replicates in each condition from three independent experiments are expressed as the mean ± SD. ** indicates a significant difference compared with the control differentiated cells (*p* < 0.01); ^aa^ indicates a significant difference compared with 1 μM CCT (*p* < 0.01).

Oil Red O (OR) staining at the FD time-point allowed visualizing the effect of CCT on the storage of intracellular lipid in differentiated 3T3-L1 adipocytes. OR staining allows estimating the amount of intracellular fat. The maximum intracellular fat detected in the control differentiated cells (CCT 0 μM) at the FD time-point was set to 100%, and the relative intracellular fat levels at the two CCT concentrations at the same time-point are depicted in Figure 2. As shown in this figure, CCT-differentiated cells at 5 μM showed a significant decrease in the content of intracellular fat (77.4 ± 11.2%, *p* < 0.01) compared to control differentiated cells. No significant effect was observed on the content of intracellular fat in cells differentiated with 1 μM CCT (97.4 ± 5.4%) or with DMSO (101 ± 10%), with respect to control differentiated cells. As expected, rosiglitazone significantly enhanced intracellular fat (219 ± 42%) as compared to control and CCT-differentiated cells (*p* < 0.001), whereas genistein significantly reduced intracellular fat (78 ± 7%) as compared to the control (*p* < 0.05). The effect of 5 μM CCT did not present a significant difference compared to genistein.

### 3.2. Crocetine Did Not Affect the Total Number of Lipid Drops or Their Size

**Figure 3:**
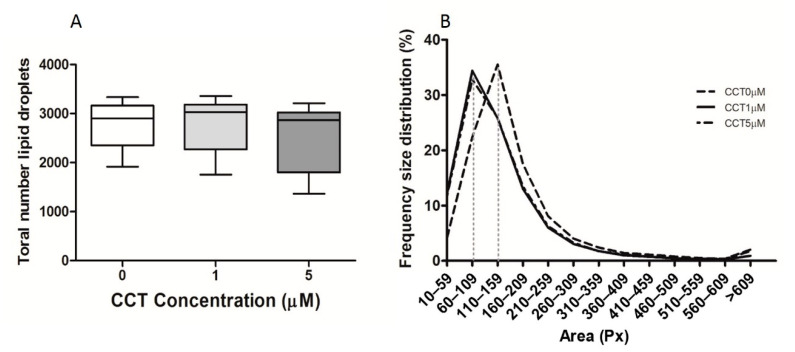
Effect of CCT on the number and size of lipid droplets. (**A**) Final lipid droplet number. Box-and-whisker plot depicting the minimum to maximum values, with the middle line representing the mean ± SD. (**B**) Intervals of frequency–area distribution. The vertical dotted line indicates the size range in which most lipid droplets were found. Droplets were analyzed at the FD time-point using WinLipid software with 1–2 photomicrographs per well from three independent experiments; 0 μM CCT (control; *n* = 15), 1 μM CCT (*n* = 11), 5 μM CCT (*n* = 10), where *n* represents the number of analyzed photomicrographs. Px, pixels.

Additionally, we evaluated whether the presence of CCT during the induction of differentiation resulted in a reduction in the number or size of lipid droplets, which could explain the lower lipid load seen in Figure 2. This was analyzed at the FD time-point using the aforementioned WinLipid software. It was observed that the induction of differentiation in the presence of the two CCT concentrations did not affect the total number of lipid droplets (Figure 3A). Regarding the size of the lipid droplets, there was no statistical difference after the induction of differentiation in the presence of CCT; however, a slight shift to the left was observed in the size distribution frequency curve. Thus, after the induction of differentiation in the presence of CCT, the highest droplet percentage was found within a smaller interval size (60–109 Px) than that of the control differentiated cells (110–159 Px) (Figure 3B).

Thus, this result indicates that the reduction in intracellular fat produced by CCT was neither due to a lower generation of lipid droplets nor a reduction in their size.

### 3.3. Crocetin Did Not Affect 3T3-L1 Cell Viability

**Figure 4:**
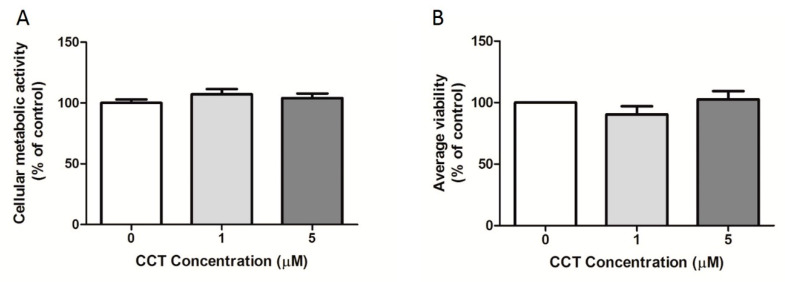
Effect of CCT on cell viability. The maximum viability detected for control differentiated cells (CCT 0 μM) was set to 100%; the relative viability of CCT-differentiated cells is shown. Values were obtained at the FD time-point. Data are expressed as the mean ± SD. (**A**) 3-(4,5-Dimethylthiazol-2-yl)-2,5-diphenyltetrazolium bromide (MTT) assay: data were obtained from at least 10 replicates in each condition from three independent experiments. (**B**) Trypan blue (TB) assay: two readings were performed per well using an automated cell counter. The percentage of viable cells (alive cells with respect to total cells) was averaged. Data were obtained from at least two replicates in each condition from three independent experiments.

In order to assess the safety of CCT, two cell viability assays were conducted at the FD time-point. Figure 4A displays the percentage cellular metabolic activity of the 3T3-L1 adipocytes obtained using the MTT assay. This assay uses metabolic activity as an indicator of cell viability by evaluating the efficiency of mitochondrial enzymes. Mitochondrial activity generates a change in color measured by spectrophotometry. The amount of color produced is proportional to the metabolic activity, thus estimating cell viability. Our results indicate no statistically significant difference in cellular viability between the control differentiated cells (CCT 0 μM) and CCT-differentiated adipocytes. Therefore, the viability of 3T3-L1 adipocytes was not affected by the concentration of CCT (1 or 5 μM: 107 ± 8% or 97 ± 8%, respectively) or DMSO (106 ± 17%). Additionally, the percentage of viable cells was determined using TB dye. Figure 4B displays the percentage of live cells with respect to total cells. Similarly, to the results obtained with MTT, no statistically significant difference in the percentage of living cells was found between the control differentiated cells (CCT 0 μM) and CCT-differentiated adipocytes. Thus, the viability of 3T3-L1 adipocytes was not affected by the concentration of CCT (1 or 5 μM: 90 ± 13% or 103 ± 13%, respectively) or DMSO (95 ± 12%); furthermore, their viability was neither affected by rosiglitazone (111 ± 8%) nor genistein (102 ± 8%).

In summary, our results indicate that neither CCT nor DMSO decreased the adipocyte mass due to cell death; thus, the decrease in intracellular fat observed in CCT-differentiated adipocytes was independent of nonspecific cell toxicity.

### 3.4. Crocetin Altered the Expression of Early and Late Genes during the Adipogenic Process

To determine the effect of CCT on the induction of the adipogenic process at a molecular level, the expression of early (*C/EBPβ* and *C/EBPδ*) and late (*PPARγ* and *C/EBPα*) genes of adipogenesis was quantified using qRT-PCR at the ID and FD time-points, respectively.

**Figure 5:**
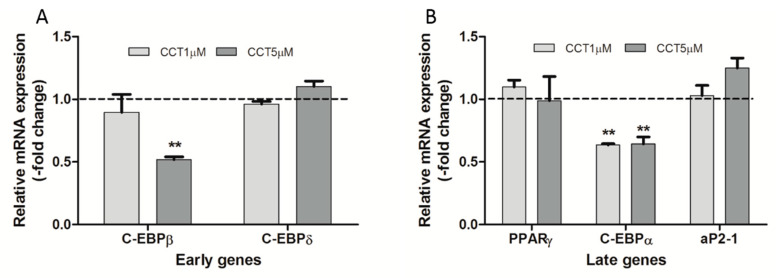
Effect of CCT on messenger RNA (mRNA) expression levels of genes regulating the adipogenic process. The relative quantitative real-time PCR (qRT-PCR) values were corrected to actin expression levels and normalized to control differentiation (0 μM CCT). Data were obtained from three independent experiments and are expressed as the mean ± SD. The maximum mRNA expression level for 0 μM CCT was set to 1 (dashed line); the relative mRNA expression levels obtained with the two CCT concentrations (1 and 5 μM) at the same time-point are depicted. (**A**) Cells were collected at the ID-time-point for the expression of early genes, CCAAT/enhancer-binding protein (*C/*EBP)β and *C/EBPδ*, evaluated using qRT-PCR with specific primer pairs. (**B**) Cells were collected at the FD time-point for the expression of late genes, peroxisome proliferator-activated receptor γ (*PPARγ*) and C/*EBPα*, evaluated using qRT-PCR with specific primer pairs; the expression of *aP2-1* (as a specific adipocyte marker) was also evaluated. ** indicates a significant difference compared with control differentiated cells (*p* < 0.01).

As shown in Figure 5 (panel A), 5 μM CCT significantly decreased *C/EBPβ* mRNA levels by 48.2% compared to control differentiated cells; however, this concentration of CCT did not alter *C/EBPδ* mRNA levels. On the other hand, 1 μM CCT modified the mRNA levels of both genes. As for the late genes, both doses of CCT (1 and 5 μM) significantly decreased *C/EBPα* mRNA levels (by 36.4% and 35.7%, respectively), without affecting the levels of *PPARγ* (Figure 5, panel B). The expression of *aP2-1* as a specific adipocyte marker was also evaluated at the FD time-point, showing no statistical difference in mRNA level with respect to control differentiated cells (Figure 5, panel B).

## 4. Discussion

In the present study, we showed that CCT could dose-dependently exert an antiadipogenic effect by decreasing lipid accumulation, a hallmark of antiobesity action. Our results showed that 5 μM CCT, but not 1 μM CCT, added at the early stage of cell differentiation reduced lipid accumulation. CCT exhibited the same efficacy as genistein in reducing intracellular fat. However, this result did not allow discriminating whether this decrease was the result of a lower generation of lipid droplets and, hence, a lower lipid load or if it was a cytotoxic effect of CCT on adipocytes via cell death, leading to a reduction in adipocyte mass and intracellular fat. To rule this out, lipid droplet metrics and cell viability were evaluated, with no differences observed in either analysis in the presence or absence of CCT.

Thus, we also investigated the effects of CCT on the expression of essential adipogenesis-related transcription factors involved in coordinating the adipogenic process. We analyzed the effect of CCT on the expression of early (*C/EBPβ* and *C/EBPδ*) and late (*C/EBPα* and *PPARγ*) genes which play key roles in adipogenesis. In this context, regulating the expression of these factors can modulate the differentiation capacity of adipocytes [43].

Typically, the mRNA levels of *C/EBPβ* and *C/EBPδ* increase during early differentiation, subsequently declining after the removal of adipogenic cocktail (AC), at which point the levels of *C/EBPα* and *PPARγ* increase [44,45]. It is known that *C/EBPβ* and *C/EBPδ* promote adipogenesis, at least in part, by inducing *C/EBPα* and *PPARγ*.

Interestingly, our results, at the ID time-point, indicate that adipocyte differentiation in the presence of the highest concentration of CCT led to a decrease in *C/EBPβ* mRNA levels, while those of C/EBPδ were not affected. However, at the FD time-point of differentiation, both tested concentrations of CCT diminished *C/EBPα* mRNA levels, while those of *PPARγ* remained unchanged.

The antiadipogenic effect of 5 μM is in line with reports showing that a knockdown of *C/EBPβ* inhibited adipocyte differentiation in 3T3-L1 preadipocytes [45]; however, in our work, CCT did not affect *C/EBPδ* expression. It is possible that an unaltered *C/EBPδ* expression can compensate for the decrease in C/EBPβ at the early stages of differentiation [46], thus playing a role in the induction of *PPARγ* expression. Most carotenoids inhibit the adipogenic process via the repression of *PPARγ* [47,48]; however, CCT did not alter *PPARγ* mRNA levels at the FD time-point. In this sense, as mentioned in Section 1, CCT is unlike β-carotene and other carotenoids, potentially exerting its effects via another mechanism [49]. On this note, it is known that β-carotene added during the differentiation of NIH 3T3-L1 preadipocytes reduced the triacylglycerol content, as well as the number and size of lipid droplets, compared with control differentiated cells by diminishing *PPARγ* expression [50]. On the other hand, Gul T et al. [51] reported that crocins at a concentration of 30 μM (treatment for 48 h) were able to diminish intracellular fat in 3T3-L1 cells, albeit producing a decrease in viability. PPARγ is not only crucial for adipogenesis but is also required for the maintenance of differentiated adipocytes [52]. It is known that knockdown of *PPARγ* compromises adipose tissue function, accompanied by insulin resistance, inflammation, angiogenesis, and fibrosis [53]. Thus, it appears that, by not altering *PPARγ* mRNA levels, CCT guarantees the culmination of the preadipocyte differentiation.

The decrease in *C/EBPβ* mRNA level seems to be CCT-dose-dependent, in contrast to that of *C/EBPα*. This suggests that CCT is an efficient carotenoid in that it selectively downregulates *C/EBPα* mRNA levels with no change in *PPARγ* expression. It has been reported that mice adipocytes in which the *C/EBPα* gene was disrupted showed defects in lipid accumulation [54]. Furthermore, various bioactive compounds reduce lipid accumulation in adipocytes by downregulating the expression of *C/EBPα* [55]. In line with these observations, the inhibition of *C/EBPα* reported herein could be involved in the decrease in intracellular fat caused by CCT during the induction of differentiation.

Taking our findings into consideration, further research should be conducted in this regard to determine the exact mechanism of CCT in combination with other standard therapeutic approaches applied to the obese population. This could contribute to the development of new strategies to improve the treatment of obesity.

Through our study, we have broadened the knowledge related to the health properties of CCT isolated from saffron as an antiadipogenic compound. Our research provides evidence that CCT efficiently reduces lipid accumulation in adipocytes, presumably via downregulation of *C/EBPα* expression. We can conclude that CCT is more potent than genistein in reducing lipid accumulation, with a similar effect produced at a lower concentration. It has been reported that genistein has a therapeutic effect on obesity [56], whereas several studies showed that its administration may be effective for adipocyte differentiation [57]. Thus, CCT’s greater efficacy than genistein suggests that this natural compound is a potential candidate to be included in dietary therapies aimed at reverting adipose tissue accumulation in obesity.

## Appendix A

**Table A1:** Mouse primer sequences (5′-3′) used for amplification.

	Forward	Reverse
**β-Actin**	AGGGAAATCGTGCGTGACAT	GGAAAAGAGCCTCAGGGCAT
**aP2**	TCACCTGGAAGACAGCTCCT	AATCCCCATTTACGCTGATG
**C/EBPβ**	TTATAAACCTCCCGCTCGGC	CTCAGCTTGTCCACCGTCTT
**C/EBPδ**	AGAACCCGCGGCCTTCTAC	GTCGTACATGGCAGGAGTCG
**C/EBPα**	CCCTTGCTTTTTGCACCTCC	TGCCCCCATTCTCCATGAAC
**PPARy**	CCAGAGTCTGCTGATCTGCG-	GCCACCTCTTTGCTCTGCTC
